# Integrating metabolomics and machine learning with in silico analysis to identify early biomarkers and molecular interactions in sepsis-associated acute kidney injury

**DOI:** 10.1038/s41598-026-45255-0

**Published:** 2026-03-27

**Authors:** Wenbo Xu, Zhouxing Zhang, Fuli Gu, Tingxian Ye, Yuechen Zhang, Wei Hu, Shaosong Xi

**Affiliations:** 1 The Fourth School of Clinical Medicine, Zhejiang Chinese Medical University, Hangzhou, 310053 China; 2 Department of Critical Care Medicine, Sir Run Run Shaw Hospital, Zhejiang University School of Medicine, Hangzhou, Zhejiang China; 3 Affiliated Hangzhou First People’s Hospital, School of Medicine, Westlake University, Hangzhou, 310006 China; 4 Department of Intensive Care Unit, Hangzhou TCM Hospital Affiliated to Zhejiang Chinese Medical University, No.1630 Huanding Road, Hangzhou, 310016 Zhejiang China

**Keywords:** Sepsis, Acute kidney injury, Metabolomics, Machine learning, Early diagnosis, Molecular docking, Biomarkers, Computational biology and bioinformatics, Diseases, Medical research, Nephrology

## Abstract

Sepsis-associated acute kidney injury (SA-AKI) presents a significant diagnostic challenge in intensive care units (ICUs), largely due to the limitations of current biomarkers. This study utilized early metabolic signatures of sepsis—specifically pre-AKI metabolic features in sepsis—to identify characteristic metabolites capable of predicting the occurrence of SA-AKI within 48 h. Using non-targeted metabolomics, serum samples from 50 sepsis patients were analyzed, including 28 patients in the SA-AKI group and 22 in the sepsis-non-AKI group. Machine learning integration of the least absolute shrinkage and selection operator (LASSO) regression and Boruta algorithms identified diagnostic metabolites. Subsequently, molecular docking was employed to explore potential metabolite-protein interactions. Among 634 detected metabolites, five key biomarkers were identified: Sebacic acid, 1-(β-D-Ribofuranosyl)-1,4-dihydronicotinamide (1-RDN), Threonic acid, Methyl acetate, and Acylcarnitine 10:2. Using leave-one-out cross-validation (LOOCV), where one patient was designated as the test set in each iteration repeated 50 times, the support vector machine (SVM) prediction model achieved an AUC value of 0.89 in the validation cohort. Molecular docking predicted stable binding between 1-RDN and phenylalanine hydroxylase (binding energy = −7.9 kcal/mol), suggesting a potential interaction and crosstalk between fatty acid metabolism and phenylalanine pathway dysregulation. This integrated metabolomics and machine learning approach, complemented by in silico molecular docking, successfully delineated early metabolic signatures of SA-AKI, provided a predictive model for early clinical intervention, and generated testable hypotheses regarding the molecular interactions linking metabolic dysregulation to renal injury.

**Supplementary Information:**

The online version contains supplementary material available at 10.1038/s41598-026-45255-0.

## Introduction

SA-AKI represents a life-threatening complication in critically ill patients, affecting nearly half of all sepsis cases^1^. The development of SA-AKI not only substantially worsens clinical outcomes but also leads to prolonged hospitalization and increased healthcare costs. Current diagnostic reliance on serum creatinine—a marker with a well-documented delayed response—often results in missed opportunities for timely intervention. There is therefore an urgent need for more sensitive approaches to enable early detection and improve patient prognosis.

Although emerging biomarkers such as kidney injury molecule-1 (KIM-1), Neutrophil Gelatinase-Associated Lipocalin (NGAL), and Liver Fatty Acid Binding Protein (L-FABP) have shown promise in SA-AKI prediction^2–4^, their diagnostic performance remains limited, particularly in the earliest phases of renal injury. This critical gap underscores the necessity for novel strategies that can capture the incipient metabolic disturbances preceding overt kidney dysfunction.

Metabolomics has emerged as a powerful tool for characterizing disease-related metabolic alterations, offering both high sensitivity and the ability to process complex, high-dimensional data^5^. When combined with machine learning—which excels at feature selection and predictive modeling—this approach holds exceptional potential for identifying robust early biomarkers. Furthermore, molecular docking provides a valuable means to explore the functional implications of metabolite-protein interactions, offering insights into underlying pathological mechanisms.

In this study, we integrated metabolomics with machine learning to systematically identify novel SA-AKI biomarkers and develop a clinically actionable prediction model. By elucidating early metabolic signatures and their mechanistic relevance, our work aims to transform SA-AKI diagnosis and pave the way for targeted therapeutic strategies.

## Results

### Characteristics of the study population

The study cohort comprised 50 ICU patients with sepsis, divided into two groups based on the occurrence of acute kidney injury within 48 h: 28 patients with SA-AKI (SA-AKI group) and 22 sepsis patients without AKI (sepsis-non-AKI group). Baseline data are shown in Table 1. The two groups exhibited no significant differences in baseline demographic characteristics (including age [P = 0.551] and gender distribution [P = 0.707]), demonstrating comparability. Significant intergroup differences were observed in multiple parameters: creatinine level within 48 h (P < 0.001), PaO₂/FiO₂ ratio (P = 0.007), plasma TNF-α levels (P = 0.038), diabetes (P = 0.045), tumor (P = 0.003), continuous renal replacement therapy (CRRT) application (P = 0.029), and SOFA score (P = 0.013). However, when these variables were simultaneously included in a multivariate logistic regression model for adjustment analysis, the aforementioned associations no longer reached statistical significance (all P > 0.05) (Supplementary Tables S1, S2).

**Table 1:** Clinical information of study cohorts.

Variable	Overall N = 50	Sepsis N = 22	SA-AKI N = 28	p-value^1^
Male, n (%)				0.707
Female	19 (38%)	9 (41%)	10 (36%)	
Male	31 (62%)	13 (59%)	18 (64%)	
Age, M (IQR)	74.50 (64.00,86.00)	73.00(65.00,87.00)	75.00(63.00,85.50)	0.551
WBC, M (IQR)	12.75 (7.30,18.50)	12.15(9.40,15.60)	13.45(6.50,18.80)	0.646
Hct, M (IQR)	0.34 (0.28,0.38)	0.35(0.31,0.40)	0.32(0.24,0.37)	0.059
MAP, M (IQR)	80.67 (75.00,94.33)	83.50(76.33,95.33)	78.50(71.00,90.67)	0.072
PLT, M (IQR)	147.00 (100.00,195.00)	149.00(118.00,230.00)	144.00(74.00,187.00)	0.305
CRP, M (IQR)	138.55 (88.60,240.00)	136.20(88.60,215.80)	170.00(91.30,261.75)	0.529
TBIL, M (IQR)	18.55 (9.60,29.00)	19.05(10.80,29.60)	18.45(9.50,27.40)	0.551
Cr_Initial, M (IQR)	97.00 (88.25,103.00)	95.00 (84.50,102.25)	98.50 (93.25,103.00)	0.089
Cr_48h, M (IQR)	142.00 (115.00,202.00)	102.50 (92.75,115.00)	167.00 (145.25,271.75)	< 0.001
PO_2_/FiO_2_, M (IQR)	283.28 (234.00,378.55)	336.30(274.50,425.20)	261.60(216.88,309.97)	0.007
Lac, M (IQR)	1.91 (1.32,2.78)	1.91(1.32,2.70)	1.94(1.34,2.89)	0.990
DM, n (%)	14 (28%)	3 (14%)	11 (39%)	0.045
Hypertension, n (%)	25 (50%)	9 (41%)	16 (57%)	0.254
CVD, n (%)	10 (20%)	3 (14%)	7 (25%)	0.480
COPD, n (%)	13 (26%)	5 (23%)	8 (29%)	0.640
CKD, n (%)	13 (26%)	4 (18%)	9 (32%)	0.264
Cirr, n (%)	5 (10%)	2 (9.1%)	3 (11%)	> 0.999
Tumor, n (%)	10 (20%)	9 (41%)	1 (3.6%)	0.003
TNF, M (IQR)	1.05(0.67,1.89)	0.84(0.53, 1.38)	1.18(0.74,2.23)	0.038
IL-2, M (IQR)	1.88(0.81, 2.35)	2.22(1.44,3.51)	1.80(0.75,2.31)	0.197
IL4, M (IQR)	1.27(0.98,1.52)	1.12(0.73,1.59)	1.29(1.12, 1.51)	0.792
IL-6, M (IQR)	284.74(125.38,2181.33)	307.63(97.09,3876.39)	280.39(164.94,1312.19)	0.732
IL-10, M (IQR)	19.97(7.62,79.49)	19.97(5.06,57.50)	19.70(8.94,108.10)	0.274
IFN-γ, M (IQR)	1.38(0.63,4.05)	1.04(0.51,6.11)	1.38(0.75,3.32)	0.883

^1^Two-tailed p values < 0.05 were considered statistically significant.

*M* median, *WBC* white blood cell, *Hct* hematocrit, *MAP* mean arterial pressure, *PLT* platelet, *CRP* C-reactive protein, *TBIL* total bilirubin, *Cr_Initial* creatinine initial, *Cr_48H* creatinine level within 48 H, *Lac* lactic acid, *DM* diabetes mellitus, *CVD* cardiovascular disease, *COPD* chronic obstructive pulmonary disease, *CVD* cardiovascular disease, *Cirr* cirrhosis, *CKD* chronic kidney disease, *TNF* tumor necrosis factor.

### Metabolomic profiling of serum samples

**Fig. 1:**
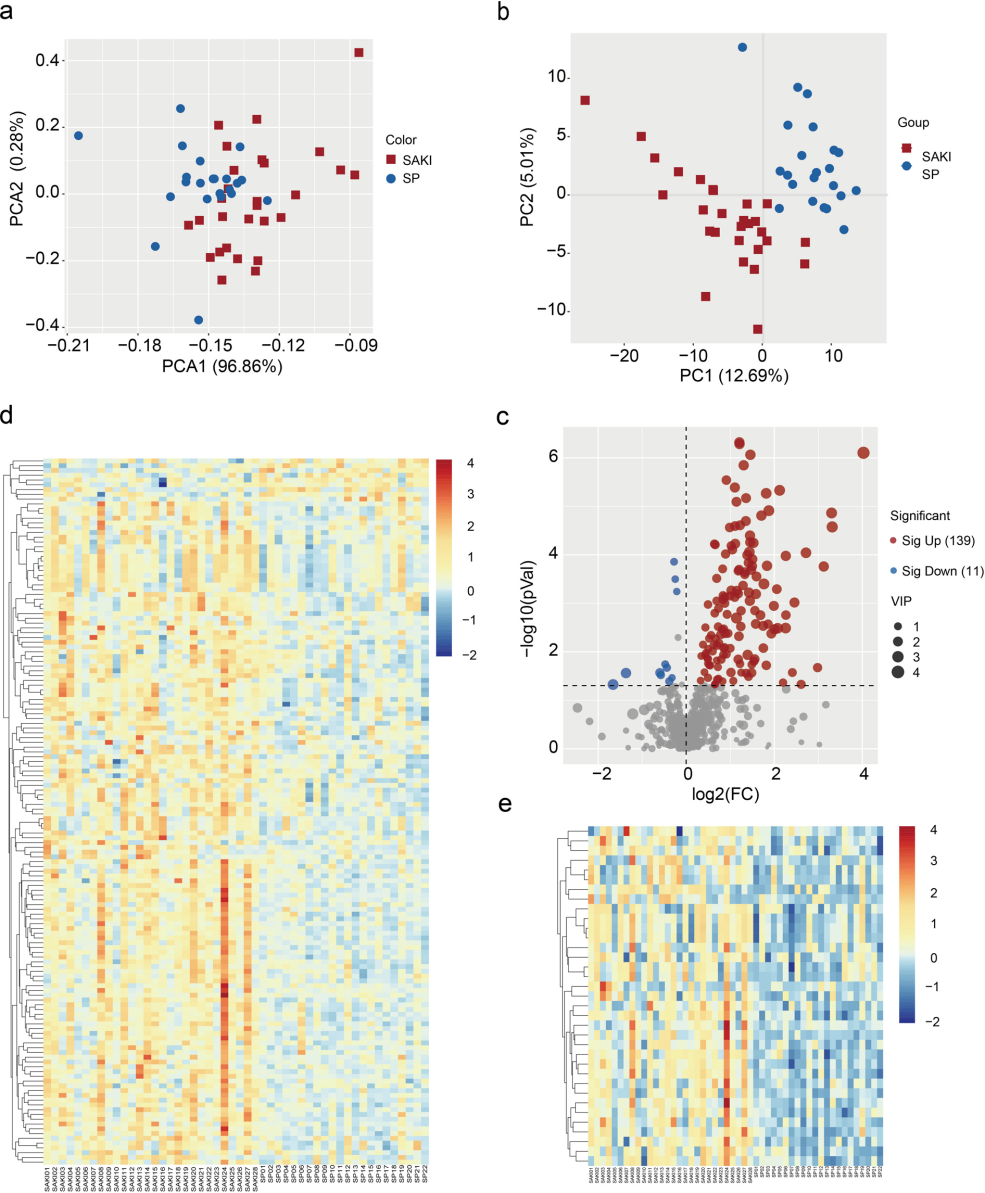
Analysis of metabolomics data between SA-AKI and SP groups. (**a**) PCA plots of 634 secondary metabolites between SA-AKI and SP groups. (**b**) OPLS-DA plots of 634 secondary metabolites between two groups. (**c**) Volcano plots of metabolites of 634 secondary metabolites between the two groups. (**d**) Heatmap of 150 secondary metabolites with statistically significant differences (P < 0.05). (**e**) Heatmap of 35 secondary metabolites (further screened for 150 secondary metabolites with P < 0.05, FC > 2 or < 0.05, VIP value > 1).

Untargeted metabolomic profiling of serum samples identified 1,425 metabolite features, with 943 showing significant upregulation and 482 demonstrating downregulation in the SA-AKI group compared to the sepsis-non-AKI group (p value < 0.05). Following secondary metabolite annotation, 634 metabolites were confidently identified. Principal Component Analysis (PCA) revealed clear separation between the SA-AKI and sepsis-non-AKI groups (Fig. 1a). Further Orthogonal Partial Least Squares-Discriminant Analysis (OPLS-DA) enhanced the differentiation between the groups (Fig. 1b), and the model showed good fit and predictive power, supporting a highly recognizable characteristic difference in metabolite levels between the SA-AKI group and the sepsis-non-AKI group. Differential analysis identified 150 significantly altered metabolites (P value < 0.05) between the two groups, comprising 139 upregulated and 11 downregulated species (Fig. 1c). Hierarchical clustering analysis of the 150 differential metabolites showed distinct grouping patterns between the SA-AKI and sepsis-non-AKI cohorts (Fig. 1d). Stringent filtering (P value < 0.05, fold change (FC) ≥ 2 or ≤ 0.5, variable importance in projection (VIP) > 1) yielded 35 potential biomarker metabolites, and their distinct expression patterns between groups were visualized in a heatmap (Fig. 1e).

**Fig. 2:**
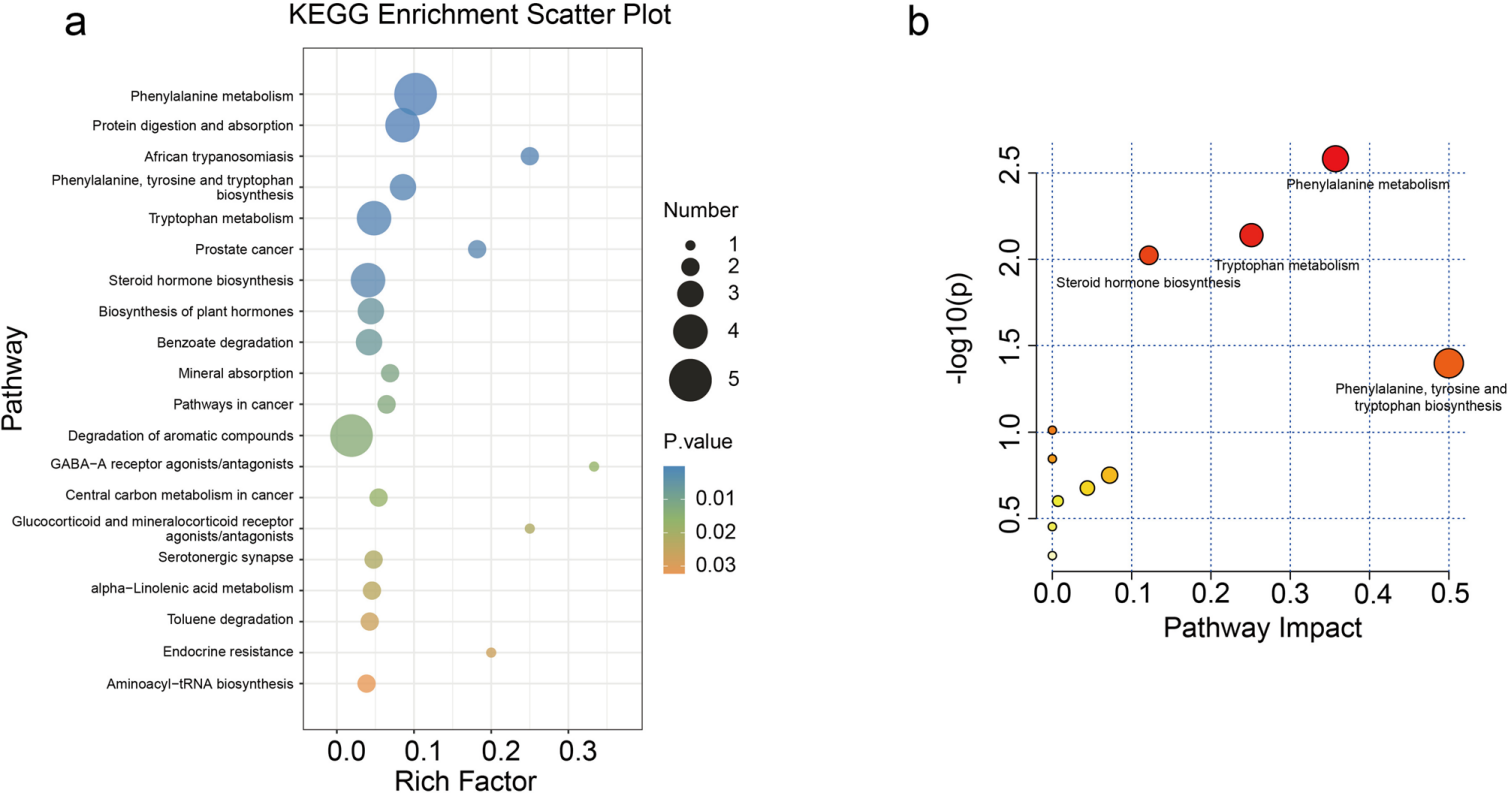
KEGG enrichment analysis of 35 secondary metabolites. (**a**) Scatter plot of KEGG enrichment. (**b**) The bubble plots of the results of KEGG pathway analysis.

KEGG pathway enrichment analysis of the differential metabolites revealed significant alterations in multiple metabolic pathways between the study groups (P < 0.05). The most prominently affected pathways included phenylalanine metabolism, protein digestion and absorption, African trypanosomiasis, phenylalanine/tyrosine/tryptophan biosynthesis, and tryptophan metabolism (Fig. 2a). Notably, the phenylalanine metabolism pathway demonstrated a particularly strong association with SA-AKI pathogenesis, as evidenced by its high pathway impact value (approximately 0.4) (Fig. 2b). This finding underscores the potential central role of phenylalanine metabolic dysregulation in SA-AKI development.

**Fig. 3:**
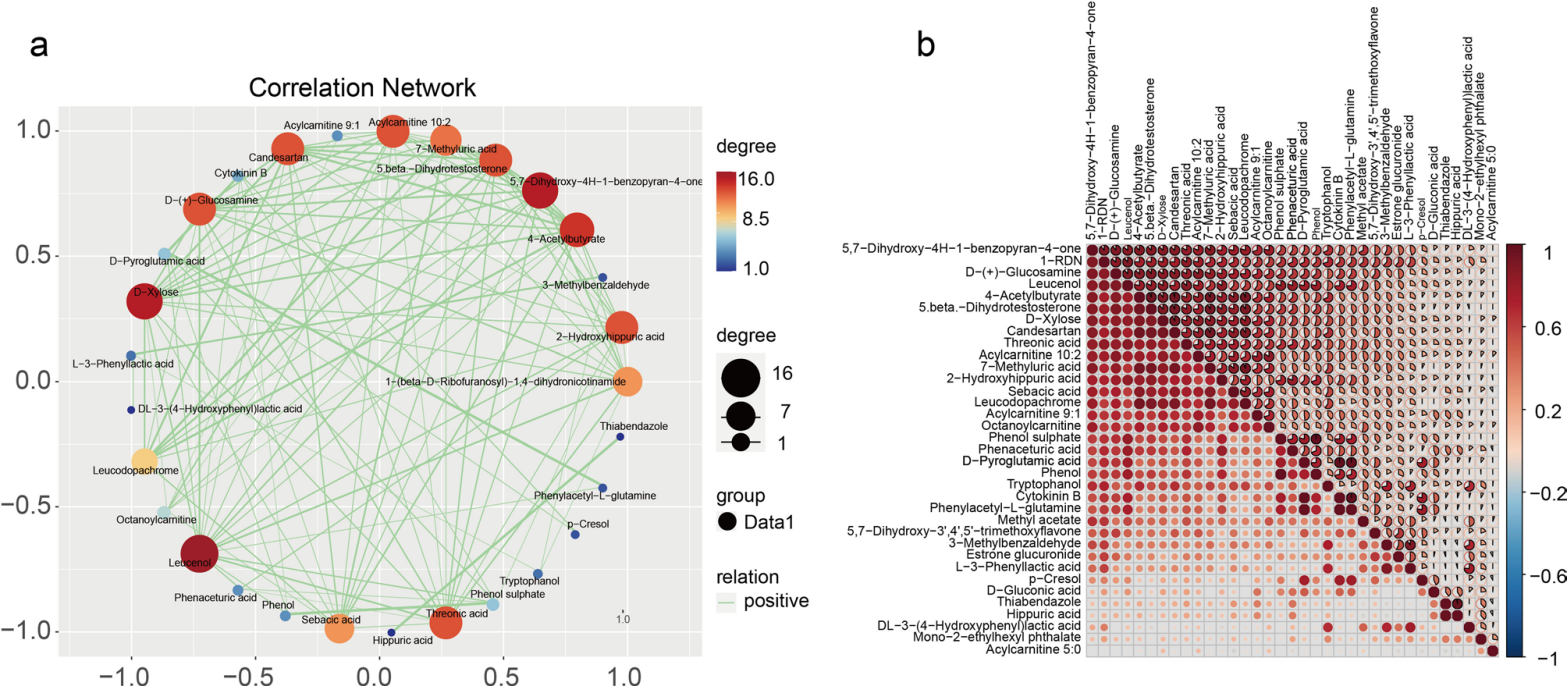
Correlation analysis of 35 secondary metabolites. (**a**) Correlation analysis network diagram. (**b**) Correlation heat map derived from Spearman correlation analysis.

Through comprehensive correlation analysis of the differential metabolites, we constructed metabolite-metabolite interaction networks to elucidate their potential functional relationships. The correlation network diagram (Fig. 3a) revealed several key metabolites—including Leucenol, D-Xylose, 5,7-Dihydroxy-4H-1-benzopyran-4-one, and Threonic acid—that exhibited particularly strong connectivity within the metabolic network. These highly interconnected metabolites likely represent critical nodes in the dysregulated metabolic pathways associated with SA-AKI. For instance, Threonic acid, as a vitamin C metabolite, and D-Xylose, which maintains pentose phosphate pathway activity, appear to play central roles in the observed metabolic disturbances. Complementary analysis using correlation coefficient heatmaps (Fig. 3b) further quantified the interaction strengths between significantly altered metabolites. Together, these findings not only reveal novel molecular mechanisms underlying SA-AKI pathogenesis but also identify promising metabolic targets for early diagnostic and therapeutic development.

### Characteristic metabolites significantly different between SA-AKI and SP groups

**Fig. 4:**
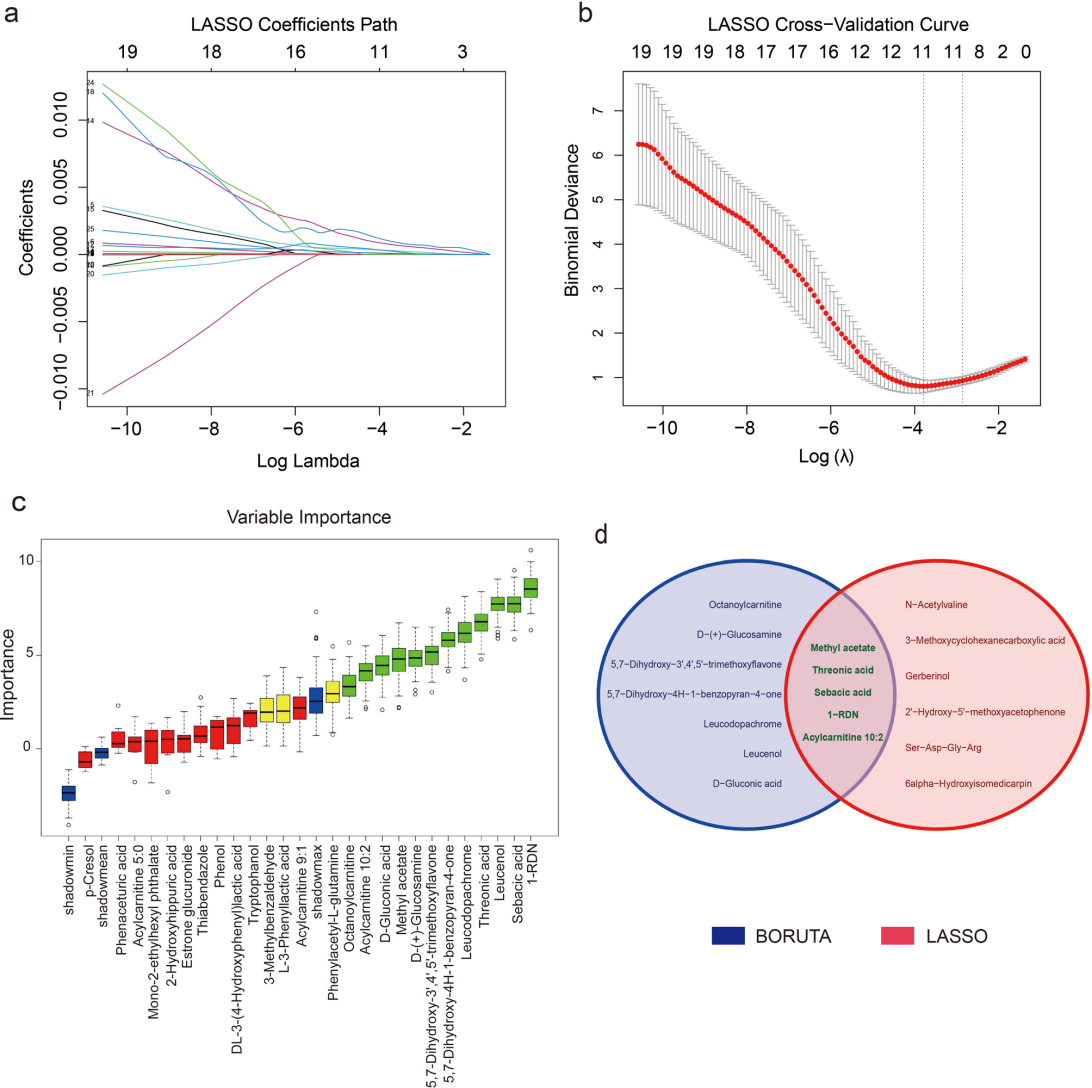
Characterization screening of 35 differential metabolites. (**a**) LASSO coefficient path diagram. (**b**) LASSO cross-validation plot. (**c**) Boruta variable significance plot. (**d**) Venn diagram. Intersection of LASSO and Boruta results common to both for 5 secondary metabolites (Sebacic acid, 1-RDN, Threonic acid, Methyl acetate, Acylcarnitine 10:2).

Utilizing the identified differential metabolites, we constructed an SA-AKI prediction model by integrating machine learning approaches. Initial preprocessing excluded 9 metabolites exhibiting low variance and high correlation. Subsequent analysis identified 11 feature metabolites through LASSO regression (Fig. 4a, b) and 12 via the Boruta algorithm (Fig. 4c). Integration of both methods via Venn analysis (Fig. 4d) yielded five key metabolites: Sebacic acid, Threonic acid, Methyl acetate, and Acylcarnitine 10:2, 1-(β-D-Ribofuranosyl)-1,4-dihydronicotinamide (1-RDN), the chemical structures of the five characteristic metabolites are detailed in Supplementary Fig. 1.

**Fig. 5:**
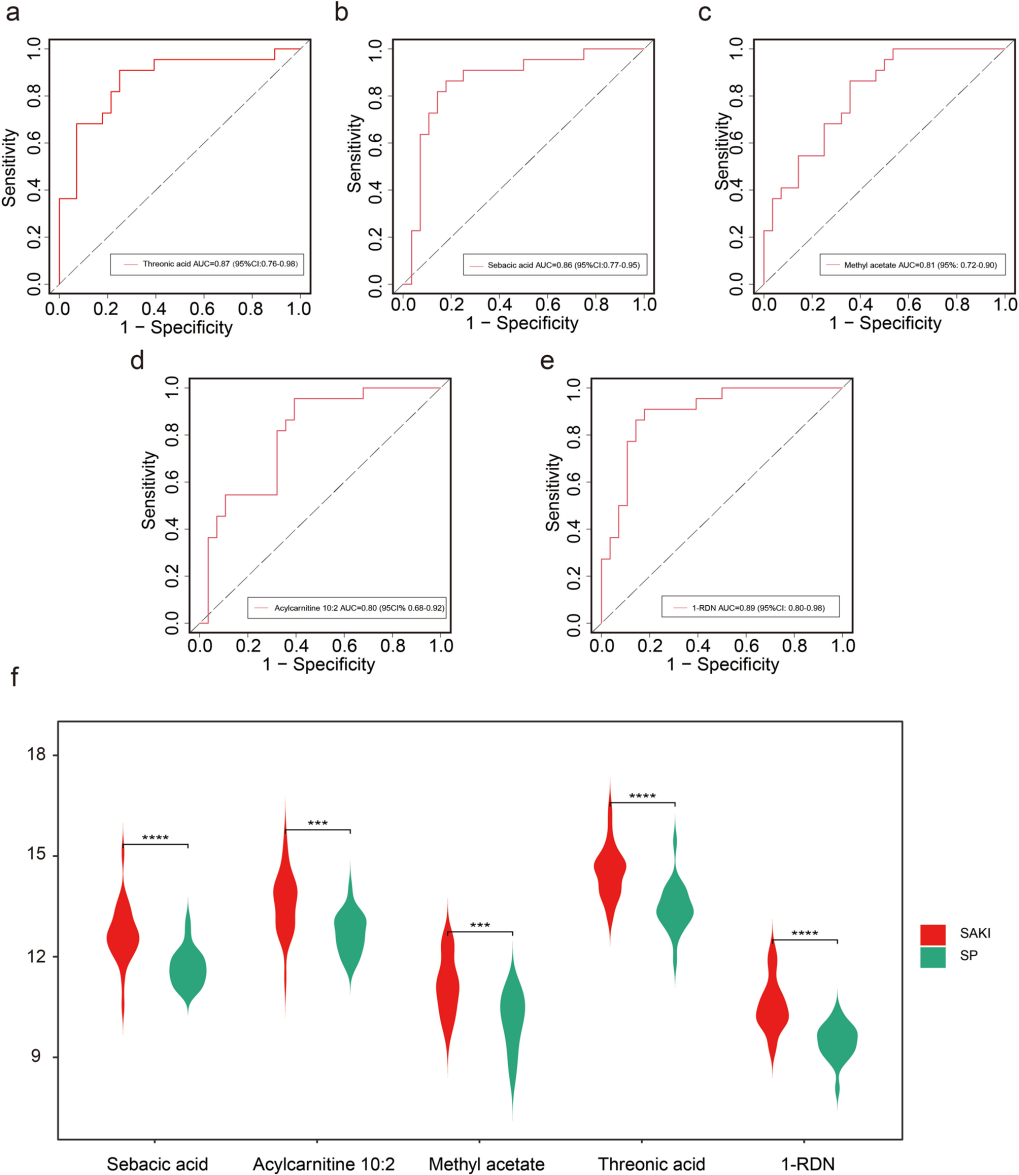
ROC plots and violin plots for the characterized variables (**a**). ROC plot for Threonic acid. (**b**). ROC plot for Sebacic acid. (**c**). ROC plot for Methyl acetate. (**d**). ROC plot for Acylcarnitine 10:2. (**e**). ROC plot for 1-RDN. (**f**). Violin plots for 5 characterized variables.

Single metabolite biomarker analysis of these five metabolites showed that each was highly discriminatory for SA-AKI, with ROC curve analysis revealing AUC values between 0.80 and 0.89 (Fig. 5a-e). Of these, 1-RDN demonstrated the best diagnostic performance (AUC = 0.89). Violin plots confirmed that all five metabolites were significantly elevated in patients with SA-AKI compared to the sepsis-non-AKI group (Fig. 5f), and the complete biochemical profile is detailed in Table 2. For further details, please refer to Supplementary Table S3.

**Table 2:** Key differential abundance metabolites between the SA-AKI and SP groups.

Metabolites	FC	P value	VIP	Regulation
Sebacic acid	2.20	8.05214E-06	2.50	Up
Acylcarnitine 10:2	2.07	7.8114E-05	2.30	Up
Methyl acetate	2.16	5.89693E-05	2.55	Up
Threonic acid	2.15	4.08178E-06	2.67	Up
1-RDN	2.31	4.77599E-07	2.75	Up

These findings highlight the translational potential of these characteristic metabolites for early SA-AKI detection and establish a foundation for developing noninvasive diagnostic approaches. Furthermore, their marked alterations provide mechanistic insights into SA-AKI pathophysiology, warranting further investigation of their biological roles and clinical utility.

### Construction and validation of an early prediction model for SA-AKI based on metabolomic features

To maximize the utilization of limited samples and obtain robust performance estimates, this study employed a LOOCV strategy. Five machine learning prediction models were constructed based on the five characteristic metabolites: Logistic Regression (LR), Extreme Gradient Boosting (XGBoost), Random Forest (RF), Support Vector Machine (SVM), and K-Nearest Neighbors (KNN).

**Fig. 6:**
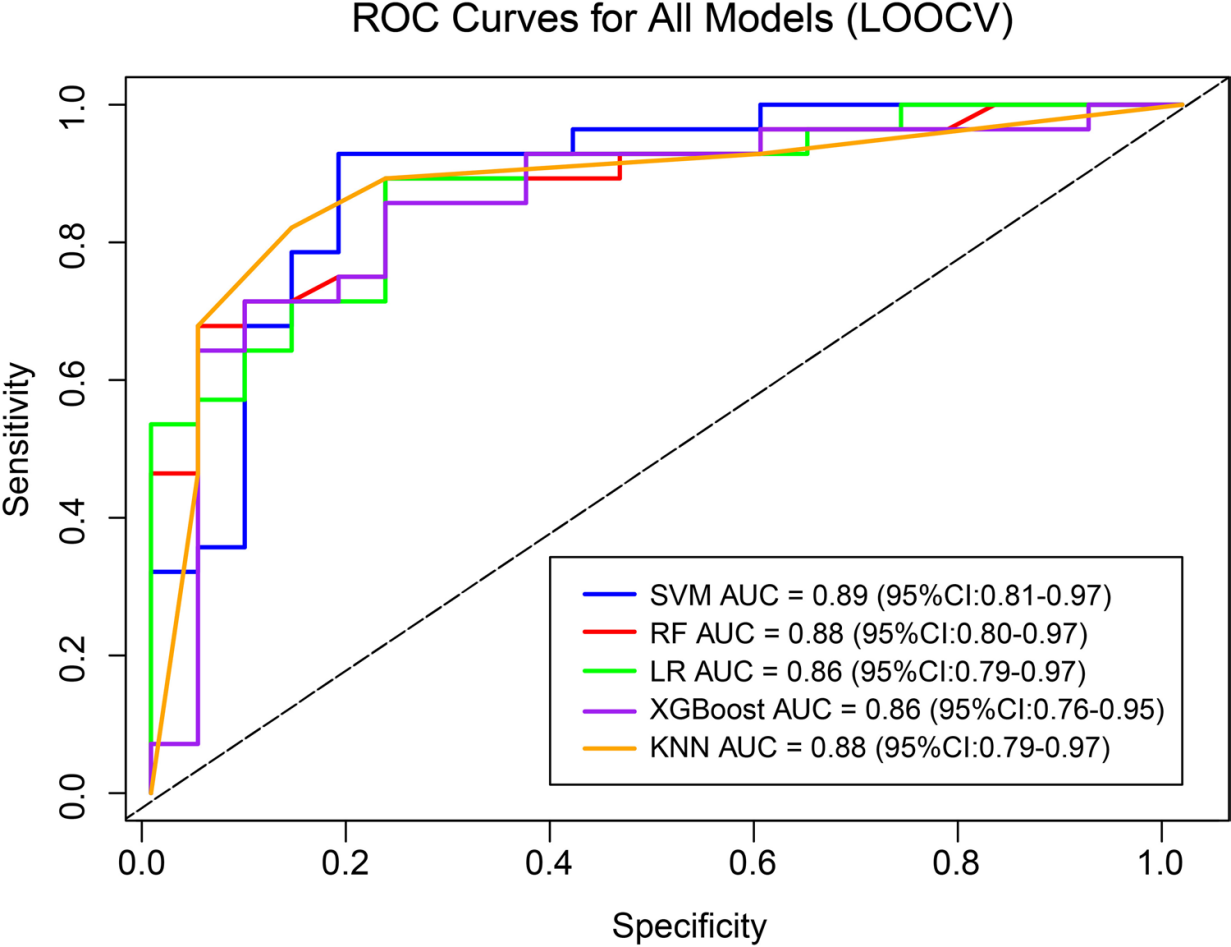
ROC curves demonstrating the discriminative ability of machine learning models for SA-AKI prediction. The SVM, RF, LR, XGBoost, and KNN models were built from a five-metabolite panel and rigorously validated using LOOCV. The AUC values (SVM: 0.89 (95% Cl 0.81–0.97); RF: 0.88 (95% Cl 0.80–0.97); LR: 0.86 (95% CI 0.79–0.97); XGBoost: 0.86 (95% Cl 0.76–0.95); KNN: 0.88 (95% CI 0.79–0.97)).

Among them, the SVM model performed best, with an AUC value of 0.89 (95% CI: 0.81–0.97, P < 0.001), and Accuracy, Sensitivity, and Specificity of 0.86 (95% CI: 0.76–0.96), 0.89 (95% CI: 0.79–0.99), and 0.86 (95% CI: 0.75–0.97), respectively (Fig. 6, Table 3). Compared to other models, SVM achieved the optimal overall performance balance while maintaining high sensitivity.

**Table 3:** Performance evaluation metrics for leave-one-out cross-validation models.

Model	AUC	Accuracy	Sensitivity	Specificity	F1	P-value^1^	P-value^2^
SVM	0.89 (0.81–0.97)	0.86 (0.76–0.96)	0.89 (0.79–0.99)	0.86 (0.75–0.97)	0.88 (0.77–0.99)	< 0.001	< 0.001
RF	0.88 (0.80–0.97)	0.84 (0.72–0.96)	0.86 (0.73–0.99)	0.82 (0.70–0.94)	0.85 (0.71–0.99)	< 0.001	< 0.001
LR	0.86 (0.79–0.97)	0.83 (0.70–0.93)	0.85 (0.73–0.97)	0.81 (0.69–0.93)	0.84 (0.70–0.98)	< 0.001	< 0.001
XGBoost	0.86 (0.76–0.95)	0.82 (0.70–0.94)	0.82 (0.68–0.96)	0.82 (0.68–0.96)	0.83 (0.68–0.97)	< 0.001	< 0.001
KNN	0.88 (0.79–0.97)	0.84 (0.72–0.96)	0.86 (0.71–1.00)	0.80 (0.70–0.90)	0.85 (0.71–0.98)	< 0.001	< 0.001

P-value^1^: DeLong Test: Significance comparison between AUC and 0.5 (random guessing).

P-value^2^: Permutation Test: Calculation of empirical p-values through 1000 random label permutations.

Importantly, the AUC values of all models were significantly higher than the random guessing level (DeLong test, P < 0.001; Permutation test, P < 0.001), confirming that the predictive capability of the selected metabolite combinations was not incidental.

### Molecular docking of characterized metabolites with acute kidney injury-associated proteins

Among the tested metabolites, 1-RDN demonstrated particularly strong binding capacity with multiple disease-associated proteins. Most notably, 1-RDN exhibited a high-affinity interaction with PAH, with a binding energy of -7.9 kcal/mol, indicative of a stable and potentially biologically relevant molecular complex^6^. The comprehensive molecular docking results, including detailed binding affinities and interaction patterns, are presented in Supplementary Table S4.

**Fig. 7:**
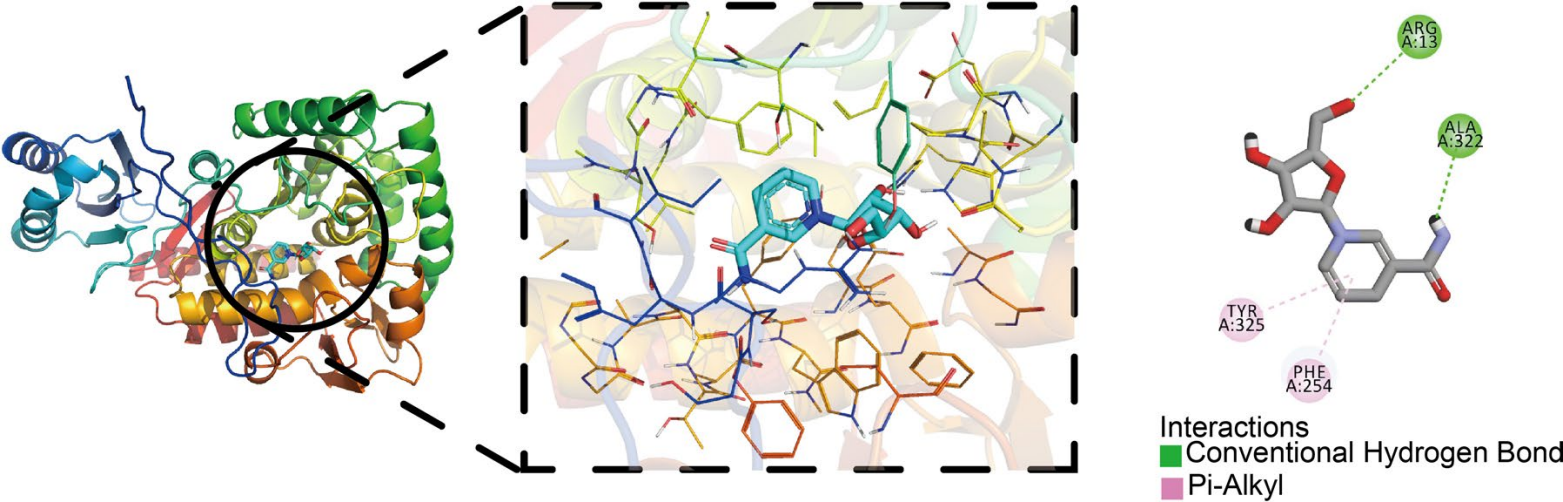
Molecular docking diagram of 1-RDN and PAH.

Additionally, residues ARG13 and ALA322 of PAH formed conventional hydrogen bonds with the ligand, HIS285 formed a Pi-Donor hydrogen bond, while PHE254 and TYR325 formed Pi-Alkyl interactions with the ligand (Fig. 7). These molecular docking results revealed a detailed binding pattern between 1-RDN and PAH, providing important clues to elucidate the molecular mechanism of this metabolite in SA-AKI. In addition, 1-RDN showed significant binding ability to other related proteins, suggesting that it may play multiple regulatory roles in the pathophysiological process of SA-AKI.

## Discussion

SA-AKI represents the predominant form of AKI in intensive care settings, comprising approximately 50% of all cases^7^. The intricate pathophysiology and limited treatment options for SA-AKI pose significant clinical challenges, substantially worsening outcomes in septic patients. This cohort study included 50 patients with sepsis, grouped based on whether acute kidney injury (AKI) developed within 48 h of diagnosis. The two groups showed no statistically significant differences in key demographic characteristics (age, gender distribution), demonstrating good balance between the groups. Although differences existed in factors such as SOFA score, PaO₂/FiO₂ ratio, diabetes prevalence, tumor status, and TNF-α levels, multivariate analysis revealed no significant independent associations. This may be attributed to multicollinearity among variables and the relatively limited sample size of the study.

Metabolomics offers exceptional sensitivity for detecting subtle alterations in metabolites and biological pathways across biofluids, cells, and tissues, thereby elucidating fundamental disease mechanisms^8^. In sepsis, this approach reveals profound disruptions to immune homeostasis^9^ and metabolic networks, including dysregulation of lipid metabolism, amino acid processing, and energy pathways resulting from systemic environmental changes^10,11^. Existing evidence demonstrates impaired mitochondrial fatty acid β-oxidation during AKI^12^, while animal metabolomic studies specifically associate SA-AKI with perturbations in central carbon/choline metabolism^13^, nucleotide metabolism, amino acid cycling, and nicotinamide pathways^14^.

These collective findings underscore the critical need to elucidate metabolic dysregulation in AKI and identify predictive biomarkers for early SA-AKI detection to inform clinical decision-making. Metabolomics datasets exhibit inherent high-dimensionality and high-throughput characteristics that are particularly amenable to machine learning approaches. In contrast to conventional analytical approaches, machine learning facilitates automated feature selection that: (i) identifies metabolites with the strongest clinical correlations; (ii) removes non-informative or duplicative features; and (iii) consequently optimizes model performance through improved predictive accuracy and enhanced generalizability.

Our study identified significant perturbations in several key metabolic pathways, most notably phenylalanine metabolism, phenylalanine/tyrosine/tryptophan biosynthesis, and tryptophan metabolism. These findings align with previous reports demonstrating marked alterations in renal cortical phenylalanine metabolism during septic AKI^15^. As an essential aromatic amino acid, phenylalanine undergoes PAH-mediated conversion to tyrosine, a critical process governing both energy homeostasis and immune regulation^16,17^. The tryptophan metabolic pathway similarly exhibited profound changes in SA-AKI. Tryptophan metabolism contributes to multiple protective mechanisms, including oxidative stress mitigation, immune response modulation, and inflammatory regulation^18^. Under physiological conditions, hepatic Tryptophan 2,3-dioxygenase (TDO) serves as the primary regulator of tryptophan catabolism. However, during AKI, a characteristic metabolic shift has been observed, characterized by suppressed TDO activity and concomitant upregulation of extrahepatic indoleamine-2,3-dioxygenase (IDO). This adaptation results in distinct alterations in kynurenine pathway metabolites (including kynurenic acid and NAD +) and enhanced anti-inflammatory responses^19–21^. These coordinated changes in aromatic amino acid metabolism—encompassing phenylalanine, tyrosine, and tryptophan pathways—highlight their collective importance in SA-AKI pathogenesis. The observed metabolic adaptations likely represent both compensatory mechanisms and potential therapeutic targets for AKI management.

Building upon the 35 significantly altered metabolites identified through initial screening, we employed a dual-algorithm approach combining LASSO regression and Boruta to refine biomarker selection. This strategy yielded five optimal metabolites: 1-RDN (an NAD + precursor), threonic acid, sebacic acid, methyl acetate, and acylcarnitine 10:2. All compounds demonstrated strong discriminatory capacity (AUC > 0.80). Based on the selected biomarkers, we employed LOOCV—a rigorous strategy suitable for small sample sizes—to construct and evaluate predictive models. This iterative training and validation approach across the entire cohort minimized random biases arising from data partitioning, thereby providing a more robust estimate of model generalization ability^22^. The five selected metabolites demonstrated excellent predictive performance across different predictive models, indicating that their ability to predict the occurrence of SA-AKI within 48 h in sepsis patients is not coincidental. This suggests they may represent precursor metabolic signatures for SA-AKI. Among the compared machine learning algorithms, Support Vector Machine (SVM) demonstrated the best classification performance (AUC = 0.89). This clearly surpasses the predictive capability of baseline creatinine levels and TNF-α (Supplementary Fig. 2). In the multivariate logistic regression analysis, when the SVM model was combined with clinically significant variables identified in univariate analysis, only the SVM model retained significant independent predictive value, while all conventional clinical parameters failed to demonstrate statistical significance. This finding further indicates that the SVM model based on five characteristic metabolites provides discriminative information beyond conventional clinical indicators (Supplementary Table S5).

As an advanced nonlinear machine learning algorithm, SVM effectively handles complex patterns in high-dimensional data, making it uniquely valuable for biomarker discovery studies such as metabolomics^23,24^. Our findings further validate this: even under the stringent LOOCV validation framework, the SVM model constructed from the five metabolites achieved significantly superior discriminative performance compared to random guessing (P < 0.001), with its predicted probabilities demonstrating good calibration against observed outcomes. This not only demonstrates the feasibility of combining metabolomics with machine learning for screening characteristic metabolites and establishing predictive models using metabolites as predictor variables, but also validates the effectiveness of the SVM algorithm in transforming these metabolites into a stable predictive model^25^. Therefore, the SVM model constructed in this exploratory study provides a viable direction and approach for the early warning of SA-AKI.

An intriguing observation from our supplementary analyses warrants discussion. While established inflammatory (TNF-α) and clinical (SOFA score, PaO_2_/FiO_2_ ratio) markers showed significant univariate associations with SA-AKI risk (Supplementary Table 1, Supplementary Fig. 2), their predictive contributions were not independent in multivariate models that included our metabolomic signature. Specifically, in a model containing the SVM-derived metabolic risk score along with TNF-α, SOFA score, and other clinical covariates, only the metabolic score remained a statistically independent predictor (P < 0.001). This indicates that the metabolic panel captures pathophysiological information that is not redundant with conventional clinical and inflammatory markers. The metabolic alterations we identified likely reflect more direct and proximal events in cellular metabolic dysfunction preceding renal injury, whereas TNF-α represents a broader, albeit important, systemic inflammatory state. Thus, the superior performance and independence of our metabolic model underscore its potential to add a novel, actionable dimension to early risk stratification beyond what is achievable with current parameters.

The observed metabolite alterations reveal an interconnected metabolic network involving energy metabolism dysregulation, impaired fatty acid oxidation, and compromised antioxidant defenses during early SA-AKI. Enhanced NAD + biosynthesis, evidenced by 1-RDN accumulation, coincides with activated antioxidant responses reflected through elevated threonic acid levels. Concurrently, disrupted fatty acid β-oxidation manifests as significant accumulations of both acylcarnitine 10:2 and sebacic acid. Notably, methyl acetate generation appears to exacerbate this metabolic imbalance by intensifying oxidative stress while further depleting cellular energy reserves, potentially establishing a self-perpetuating cycle in SA-AKI pathogenesis.

The metabolite 1-RDN represents a reduced form of nicotinamide riboside (NRH) characterized by a β-D-ribofuranosyl moiety. As a crucial intermediate in NAD + biosynthesis, NRH contributes to this essential cofactor’s roles in cellular metabolism, DNA repair, and energy generation. Impaired NAD + biosynthesis has been established as a risk factor for AKI susceptibility^26^. The kidney, ranking second only to the heart in mitochondrial density^27^, demonstrates particularly high metabolic activity in tubular epithelial cells. Under physiological conditions, NAD + serves as a mitochondrial electron carrier, supporting the high energetic demands of fatty acid β-oxidation and ATP production^28^. However, AKI induces significant NAD + depletion through both excessive consumption and diminished synthesis, leading to impaired fatty acid oxidation, renal lipid accumulation, and tubular dysfunction^28–33^. Our findings suggest that SA-AKI patients experience early oxidative and energetic stress, triggering compensatory NAD + biosynthesis pathways including 1-RDN accumulation and enhanced nicotinamide riboside (NR)-mediated NAD + generation.

Threonic acid, a metabolic derivative of ascorbic acid, reflects the activation of this potent antioxidant system. Ascorbic acid confers renal protection through multiple mechanisms, including free radical scavenging, oxidative damage mitigation, and preservation of enzymatic functions^34^. Clinical evidence demonstrates its capacity to attenuate AKI by reducing lipid peroxidation, suppressing inflammatory responses, and enhancing renal oxygen utilization^35–37^. The significant elevation of threonic acid in our SA-AKI cohort indicates substantial ascorbic acid mobilization against sepsis-induced oxidative stress, potentially serving as an early biomarker of AKI risk. Acylcarnitine 10:2, a crucial intermediate in mitochondrial fatty acid β-oxidation, has established associations with metabolic and cardiovascular disorders^38–41^. Its marked accumulation in our study implicates impaired fatty acid oxidation as a pivotal mechanism in early SA-AKI pathogenesis. Similarly, sebacic acid—a 10-carbon dicarboxylic acid and TCA cycle intermediate—accumulates during dysfunctional fatty acid oxidation and energy production^42–44^. Sepsis-induced ketogenesis exacerbates this metabolic dysregulation, as acetone accumulation intensifies oxidative stress, while its subsequent conversion to methyl acetate via acetone monooxygenase further aggravates cellular injury. Collectively, these metabolic perturbations demonstrate the profound disruption of renal energy homeostasis, antioxidant defenses, and lipid metabolism characteristic of SA-AKI, revealing an interconnected pathophysiology of oxidative stress and bioenergetic failure. The clinical implications of this study lie in shifting management strategies from passive support to active intervention. Therapeutic approaches should implement precise metabolic support, carefully evaluate nutritional strategies, and prioritize energy supply to alleviate mitochondrial fatty acid oxidation dysfunction. Concurrently, antioxidant therapy should be intensified. Finally, meticulous hemodynamic monitoring must ensure adequate renal perfusion, and the use of any nephrotoxic medications should be avoided with extreme caution to prevent cumulative injury.

Molecular docking analysis was performed to investigate the potential of 1-RDN to interact with PAH and thereby exert disease-modifying effects. The results demonstrated a strong binding affinity (ΔG = -7.9 kcal/mol). Structural analysis, conducted using the non-bonded interaction analysis tool in Discovery Studio Visualizer, revealed that 1-RDN forms a comprehensive interaction network with PAH, including conventional hydrogen bonds with ARG13 and ALA322, a Pi-Donor hydrogen bond with HIS285, and Pi-Alkyl interactions with PHE254 and TYR325. This robust in silico evidence suggests a hypothesis that 1-RDN may effectively bind to PAH and potentially influence its enzymatic activity. To transition from this computational prediction to established mechanism, future work should first employ biophysical techniques such as Surface Plasmon Resonance (SPR) or Isothermal Titration Calorimetry (ITC) to quantitatively confirm the direct binding between 1-RDN and purified PAH protein. Subsequently, establishing a causal link to renal pathology would require functional studies in cellular and animal models. For instance, in vitro experiments using renal tubular epithelial cells (e.g., HK-2) with PAH knockdown or overexpression could assess whether the putative effects of 1-RDN on injury markers (e.g., apoptosis, inflammation) are dependent on PAH expression. These would be complemented by in vivo studies in a septic AKI model, evaluating if 1-RDN administration ameliorates renal dysfunction and tubular damage, and if this protective effect is attenuated upon renal-specific PAH inhibition.

PAH serves as the rate-limiting enzyme in phenylalanine metabolism, catalyzing the conversion of phenylalanine to tyrosine, a biochemical reaction critical for maintaining normal physiological processes including neurotransmitter biosynthesis (dopamine, norepinephrine, and epinephrine) and hormone production^45^. Given the pronounced metabolic dysregulation in SA-AKI, particularly the crosstalk between fatty acid and amino acid pathways^46,47^, the potential interaction between 1-RDN and PAH proposed here offers a novel mechanistic link. We hypothesize that 1-RDN-mediated modulation of PAH activity could disrupt phenylalanine/tyrosine flux. Although PAH is a hepatic enzyme, it is also expressed in the proximal tubules of the renal cortex^48^. Despite no significant differences in baseline liver function among patients in this study, a marked disparity in phenylalanine metabolism levels emerged between the two groups. Combined with molecular docking results, this further guides us toward considering a complementary hypothesis that transcends the classical ‘hepatic center’ model: Beyond systemic hepatic PAH inhibition, localized renal disruption of aromatic amino acid metabolism may play a more direct “final blow” role in the pathogenesis of SA-AKI, exacerbating immune and metabolic dysfunction in SA-AKI. In summary, our study posits the "1-RDN-PAH" axis as a plausible and novel mechanistic hypothesis in SA-AKI. While this model provides a conceptual framework for targeting metabolic regulation in critical illness, its validation through the aforementioned experimental approaches is a crucial next step.

By integrating untargeted metabolomics and machine learning, this study characterized early metabolic disturbances in SA-AKI and identified five key metabolites: Sebacic acid, 1-RDN, Threonic acid, Methyl acetate, and Acylcarnitine 10:2. A SVM model based on these metabolites showed an AUC of 0.89, with sensitivity of 0.89 and specificity of 0.86, performing better than conventional biomarkers such as serum creatinine. Molecular docking indicated that 1-RDN stably binds to PAH via hydrogen bonding and Pi-alkyl interactions (binding energy = -7.9 kcal/mol). These results suggest that 1-RDN may promote renal tubular injury by exacerbating fatty acid oxidation disorders and oxidative stress through modulation of phenylalanine metabolism.

This study used early metabolic profiles from ICU sepsis patients to predict SA-AKI within 48 h. Blood was collected before AKI onset to capture initiating metabolic changes. Although LOOCV was applied, the modest sample size may still affect model stability and feature selection power. The single-center design also limits generalizability.Technically, despite standardized metabolomic protocols, batch effects or instrument drift could influence quantification. Molecular docking indicated a high-affinity interaction between 1‑RDN and PAH (binding energy: ‑7.9 kcal/mol), yet this static model cannot simulate dynamic binding processes in physiological conditions. While the five-metabolite panel showed promising clinical utility in decision‑curve analysis, further validation is essential. External testing in larger, multicenter cohorts and functional studies are needed to confirm its predictive value and the proposed 1‑RDN‑PAH interaction.

Finally, while this integrated LC–MS and machine learning approach has successfully identified promising early biomarkers for SA-AKI, we acknowledge a key limitation for immediate clinical translation: the inherent turnaround time and complexity of untargeted metabolomics profiling render it unsuitable for rapid point-of-care diagnosis. The true clinical utility of the biomarkers discovered here, such as Sebacic acid and 1-RDN, lies in their potential for development into targeted, rapid assays. Future work should focus on translating these findings into clinically feasible formats, such as antibody-based dipstick tests or simplified enzymatic assays that can deliver results at the bedside within minutes. This study provides the essential molecular targets for such development.

## Conclusion and perspectives

In summary, this study elucidated metabolic dysregulation associated with sepsis-associated acute kidney injury (SA-AKI) through an integrated approach combining metabolomics and machine learning. We identified a unique metabolic signature specific to SA-AKI and established a predictive model based on five metabolic biomarkers. Furthermore, molecular docking analysis revealed potential interactions between key metabolites and disease-associated proteins, offering new insights into the pathogenesis of SA-AKI.

Our findings highlight the 1-RDN-PAH interaction as a putative node linking metabolic dysregulation to renal injury in sepsis. This opens two strategic avenues for future mechanistic and therapeutic research: First, to investigate whether modulating the metabolic pathway involving 1-RDN (e.g., by targeting its synthesis or degradation) confers renal protection. Second, to explore the therapeutic potential of targeting the PAH protein, either by designing ligands based on the predicted 1-RDN binding site or by regulating its activity, given its newly implicated role in SA-AKI pathophysiology.

## Materials and methods

### Study population

This prospective study enrolled fifty consecutive sepsis patients admitted to the intensive care unit of Hangzhou First People’s Hospital from April 2021 to November 2022. Based on the 2012 Kidney Disease: Improving Global Outcomes (KDIGO) criteria, 28 patients developed sepsis-associated acute kidney injury (SA-AKI) within 48 h of sepsis onset, while 22 served as non-AKI controls. The median time from sepsis diagnosis to sample collection was 6.5 h, and the median time from sample collection to AKI diagnosis was 21 h.

The inclusion criteria comprised: (1) ICU admission with a sepsis diagnosis per the Sepsis-3 criteria; (2) age ≥ 18 years; (3) a minimum 72-h hospitalization. Exclusion criteria included: (1) incomplete clinical records; (2) pre-existing chronic kidney disease (stage ≥ 3) or renal transplant recipients; (3) concurrent significant renal pathologies.

This study was approved by the Ethics Committee of Hangzhou First People’s Hospital (Ethics Approval Certificate: KY-20211105–0078-01). All studies were conducted in accordance with relevant guidelines and regulations, and informed consent was obtained from all participants and/or their legal guardians.

### Sample collection and processing

Peripheral venous blood (5 mL) was collected immediately following ICU admission and confirmation of sepsis diagnosis. All AKI events were confirmed within the subsequent 48-h observation period. Blood samples were centrifuged (3000 × g, 15 min, 4 °C) to isolate serum. All samples underwent standardized preprocessing including: (1) pre-cooling on ice; (2) protein precipitation; (3) vortex mixing for 30 s; (4) incubation at 4 °C for 30 min; and (5) centrifugation at 12,000 × g for 10 min at 4 °C. Processed samples were aliquoted and stored at -80 °C until LC–MS analysis.

### Metabolomics analysis

Metabolite separation was performed using an UltiMate 3000 UPLC system (Thermo Scientific) with detection in both positive and negative ionization modes. Raw data processing included peak extraction and alignment using XCMS software (version 3.3.19), followed by quality control assessment with metaX (version 1.4.19). Data preprocessing comprised median normalization and missing value imputation.

Metabolite identification followed a two-tiered approach to ensure reliable annotation. First, features detected by XCMS were matched to the HMDB and KEGG databases with a mass accuracy threshold of < 10 ppm^49–51^. Second, to improve specificity and resolve isomers, experimental MS/MS spectra were matched against our in-house tandem MS library (curated from public spectral repositories). Matches were evaluated using a cosine similarity score (range 0–1); a score > 0.7 was considered a high-confidence spectral match. Based on this workflow, all reported metabolite identifications are assigned Level 2 confidence following established metabolomics reporting standards.

### Statistical analysis

Metabolite abundances were log2-transformed and compared between the SA-AKI and sepsis-non-AKI groups using two-tailed Student’s t-tests with Benjamini–Hochberg correction for multiple comparisons. Differential metabolites were identified based on the following criteria: (1) FC ≥ 2 or ≤ 0.5; (2) adjusted p-value < 0.05; and (3) VIP score ≥ 1 from OPLS-DA. Feature selection was performed using LASSO regression and the Boruta algorithm. All statistical analyses were conducted using R software (version 4.1.2).

### Predictive modeling

We rigorously evaluated the predictive performance of candidate metabolite combinations using LOOCV, a method that provides near-unbiased model performance estimates for small datasets. For the five machine learning algorithms (LR, XGBoost, RF, SVM, and KNN), the validation process included iteration, feature selection replication, data preprocessing, model training and prediction, and result output. This process was repeated 50 times to ensure each patient was used once as the test set. Model evaluation synthesized predictions from all LOOCV iterations to compute overall performance metrics, including the Area Under the Receiver Operating Characteristic Curve (AUC), accuracy, sensitivity, and specificity. To prevent overfitting, model hyperparameters were optimized via random search within each training fold.

### Molecular docking analysis

To investigate potential functional interactions, we performed molecular docking simulations using PyRx software to analyze binding between the identified metabolites and AKI-associated proteins. Target protein selection was guided by established pathophysiological mechanisms, focusing on four key categories: renal tubular injury markers (L-FABP)^52^, fibrosis mediators (Transforming Growth Factor Beta, TGF-β)^53^, fatty acid oxidation regulators (Acyl-CoA Dehydrogenase Long Chain, ACADL; Peroxisome Proliferator-Activated Receptor Alpha, PPARα; Medium-Chain Acyl-CoA Dehydrogenase, MCAD; AMP-Activated Protein Kinase, AMPK)^54^, and phenylalanine metabolism enzymes (Phenylalanine Hydroxylase, PAH)^55^. SDF structure files of the identified metabolites were retrieved from the PubChem database (https://pubchem.ncbi.nlm.nih.gov/), while crystal structures of the target proteins were acquired from the Protein Data Bank (PDB; https://www.rcsb.org/).Protein structures were prepared by removing water molecules and co-crystallized ligands using PyMOL 2.1.0, followed by hydrogen atom addition and Gasteiger charge assignment using AutoDock Tools 1.5.6. Molecular docking simulations were conducted using AutoDock Vina 2.0 integrated in the PyRx platform, with binding affinity (ΔG, kcal/mol) serving as the primary stability metric. Resultant complexes were visualized using PyMOL 2.1.0 for three-dimensional representation, while two-dimensional interaction diagrams were generated with Discovery Studio 2020 Client (BIOVIA) to identify specific molecular interactions.

## Supplementary Information

Supplementary Information 1.

Supplementary Information 2.

Supplementary Information 3.

## Data Availability

Untargeted metabolomics raw data have been deposited to the EMBL-EBI MetaboLights database with the identifier MTBLS12411 (https://www.ebi.ac.uk/metabolights/editor/MTBLS12411). Structural information on disease-associated proteins is available in the Protein Data Bank (PDB; https://www.rcsb.org/).
