# Supplementary figures and images for: Integrating metabolomics and machine learning with in silico analysis to identify early biomarkers and molecular interactions in sepsis-associated acute kidney injury
Source: Sci Rep. 2026 Mar 27;16:10963. doi: 10.1038/s41598-026-45255-0 (PMC13039459; doi:10.1038/s41598-026-45255-0)

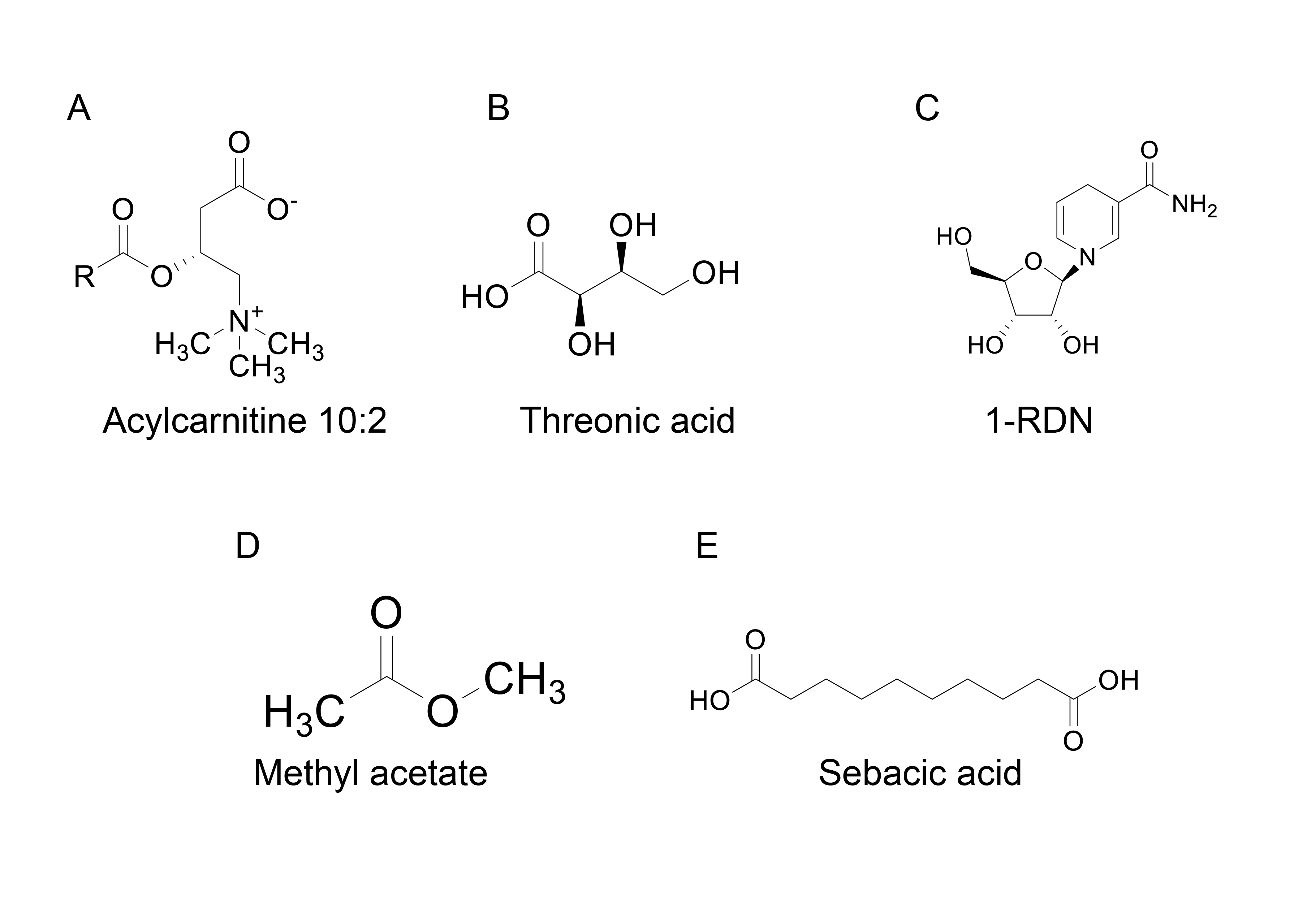

Supplement: Supplementary file 1 — Supplementary Information 1. [file 41598_2026_45255_MOESM1_ESM.tif]

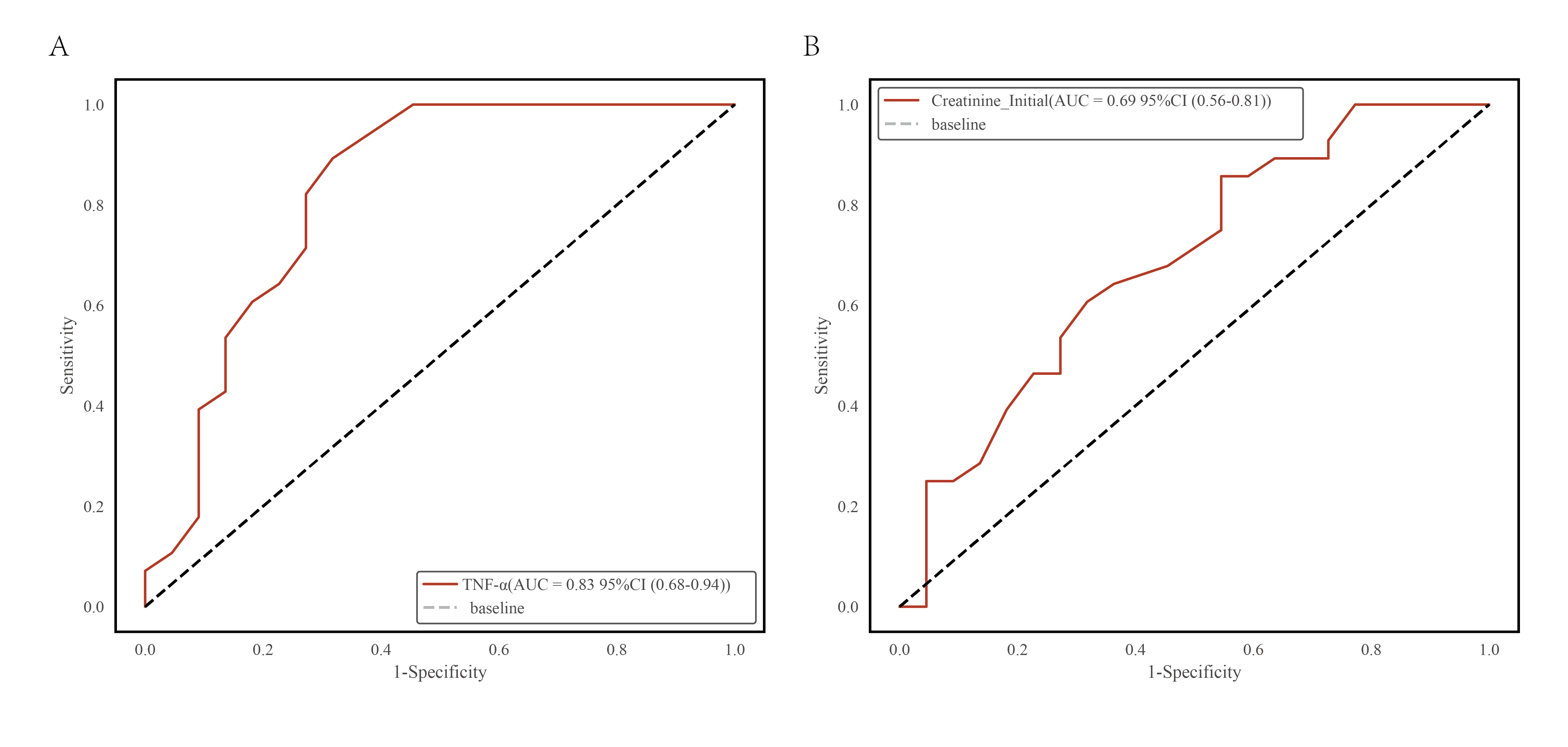

Supplement: Supplementary file 2 — Supplementary Information 2. [file 41598_2026_45255_MOESM2_ESM.tif]
