# Supplementary material for: Integrating metabolomics and machine learning with in silico analysis to identify early biomarkers and molecular interactions in sepsis-associated acute kidney injury
Source: Sci Rep. 2026 Mar 27;16:10963. doi: 10.1038/s41598-026-45255-0 (PMC13039459; doi:10.1038/s41598-026-45255-0)
Supplement: Supplementary file 3 — Supplementary Information 3. [file 41598_2026_45255_MOESM3_ESM.docx]

**Supplementary Material**

**Supplementary Methods:Untargeted Metabolomics Experiment and Data Analysis**

1.1 Metabolite extraction description

Liquid samples:

The collected samples were thawed on ice, and metabolite were extracted with 80% methanol Buffer. Briefly, 100μL of sample was extracted with 400 μL of precooled methanol. The extraction mixture was then stored in 30 min at -20°C. After centrifugation at 20000 g for 15 min, the supernatants were transferred into new tube to and vacuum dried. The samples were redissolved with 100μL 80% methanol and stored at -80°C prior to the LC-MS analysis. In addition, pooled QC samples were also prepared by combining 10 μL of each extraction mixture.

Solid samples:

The collected samples were thawed on ice, and metabolite were extracted with 80% methanol Buffer. Briefly, 50 mg of sample was extracted with 0.5 ml of precooled 80% methanol. The extraction mixture was then stored in 30 min at -20°C. After centrifugation at 20000g for 15 min, the supernatants were transferred into new tube to and vacuum dried. The samples were redissolved with 100μL 80% methanol and stored at -80°C prior to the LC-MS analysis. In addition, pooled QC samples were also prepared by combining 10 μL of each extraction mixture.

1.2 Description of liquid phase parameters

All samples were acquired by the LC-MS system followed machine orders. Firstly, all chromatographic separations were performed using an UltiMate 3000 UPLC System(Thermo Fisher Scientific, Bremen, Germany). An ACQUITY UPLC T3 column (100mm*2.1mm, 1.8µm, Waters, Milford, USA) was used for the reversed phase separation. The column oven was maintained at 40°C. The after, 5mM ammonium acetate and 5mM acetic acid) and solvent B (Acetonitrile). Low rate was 0.3 ml/min and the mobile phase consisted of solvent A. Gradient elution conditions were set as follows: 0~0.8 min, 2% B; 0.8~2.8 min, 2% to 70% B; 2.8~5.6min, 70% to 90% B; 5.6~6.4 min, 90% to 100% B; 6.4~8.0 min, 100% B; 8.0~8.1 min, 100%to 2%B; 8.1~10 min, 2%B.

1.3 Mass Spectrometry Parameter Description

A high-resolution tandem mass spectrometer TripleTOF 6600 (SCIEX, Framingham, MA, USA) was used to detect metabolites eluted form the column. The Q-TOF was operated in both positive and negative ion modes. The curtain gas was set 30 PSI, Ion source gas1 was set 60 PSI, Ion source gas2 was set 60 PSI, and an interface heater temperature was 500 ℃.For positive ion mode, the Ionspray voltage floating were set at 5000V, respectively. For negative ion mode, the Ionspray voltage floating were set at -4500V, respectively. The mass spectrometry data were acquired in IDA mode. The TOF mass range was from 60 to 1200Da. The survey scans were acquired in 150ms and as many as 12 product ion scans were collected if exceeding a threshold of 100 counts per second (counts/s) and with a 1+ charge-state. Dynamic exclusion was set for 4s.During the acquisition, the mass accuracy was calibrated every 20 samples. Furthermore, in order to evaluate the stability of the LC-MS during the whole acquisition, a quality control sample (Pool of all samples) was acquired after every 10 samples.

2 Information Analysis Process

2.1 Information Analysis Description

The acquired MS data pretreatments including peak picking, peak grouping, retention time correction, second peak grouping, and annotation of isotopes and adducts was performed using XCMS software. LC−MS raw data files were converted into mzXML format and then processed by the XCMS, CAMERA and metaX toolbox implemented with the R software. Each ion was identified by combining retention time (RT) and m/z data. Intensities of each peaks were recorded and a three dimensional matrix containing arbitrarily assigned peak indices (retention time-m/z pairs), sample names (observations) and ion intensity information (variables) was generated.

The online KEGG, HMDB database was used to annotate the metabolites by matching the exact molecular mass data (m/z) of samples with those from database. If a mass difference between observed and the database value was less than 10 ppm, the metabolite would be annotated and the molecular formula of metabolites would further be identified and validated by the isotopic distribution measurements. We also used a in-house fragment spectrum library of metabolites to validate the metabolite identidification.

Statistical analysis was performed in R (version 4.0.0). The raw protein intensity will be normalized by method "medium", Hierarchical clustering was performed using pheatmap package. Principal component analysis (PCA) was performed using metaX package. The PLSDA analysis is performed by the R package ropls and the VIP values of each variable are calculated.Correlation analysis was performed by Pearson correlation coefficient of cor package .The three conditions of P Value<0.05, difference multiple >1.2 obtained by T test and VIP calculated by PLSDAanalysis simultaneously met the screening of the final metabolites with significant differences.. Hypergeometric-based enrichment analysis with KEGG Pathway was performed to annotate protein sequences. individually.The software GSEA (v4.1.0) and MSigDB were used for gene set enrichment analysis to determine whether a set of genes in a specific KEGG pathway in different situations. Meeting this condition |NES|>1, NOM p-val<0.05, FDR q-val<0.25 were considered tobe significantly different between the two groups. The network map is drawn according to the pathway where the metabolite is located.

**Supplementary Tables**

Supplementary Table S1. Clinical information of study cohorts

| Variable | Overall N = 50 | SP N = 22 | SA-AKI N = 28 | p-value |
| --- | --- | --- | --- | --- |
| CRRT, n (%) | | | | 0.029 |
| No | 40 (80%) | 21 (95%) | 19 (68%) |  |
| Yes | 10 (20%) | 1 (4.5%) | 9 (32%) |  |
| ARDS, n (%) | | | | 0.734 |
| No | 39 (78%) | 18 (82%) | 21 (75%) |  |
| Yes | 11 (22%) | 4 (18%) | 7 (25%) |  |
| Ventilation, n (%) | | | | 0.087 |
| No | 25 (50%) | 14 (64%) | 11 (39%) |  |
| Yes | 25 (50%) | 8 (36%) | 17 (61%) |  |
| 28 days survival rate, n (%) | | | | 0.480 |
| No | 10 (20%) | 3 (14%) | 7 (25%) |  |
| Yes | 40 (80%) | 19 (86%) | 21 (75%) |  |
| 100 days survival rate, n (%) | | | | 0.525 |
| No | 16 (32%) | 6 (27%) | 10 (36%) |  |
| Yes | 34 (68%) | 16 (73%) | 18 (64%) |  |
| Sofa Score, median (IQR) | 7.00(5.00,11.00) | 5.50(4.00,7.00) | 9.00(5.50,11.50) | 0.013 |
| Apache II Score, median (IQR) | | | | |
| At admission | 17.00(12.00,22.00) | 17.00(10.00,21.00) | 17.00(13.00,22.00) | 0.604 |
| At 72 hours | 6.00(5.00,11.00) | 6.00(5.00,8.00) | 6.50(5.00,11.00) | 0.113 |
| GCS Score, n (%) | | | | 0.152 |
| 0-5 | 2 (4.0%) | 0 (0%) | 2 (7.2%) |  |
| 6-10 | 1 (2.0%) | 0 (0%) | 1 (3.6%) |  |
| 11-15 | 47 (94.0%) | 22 (100%) | 25 (89.2%) |  |

Two-tailed p values <0.05 were considered statistically significant.

Abbreviations:

CRRT, Continuous Renal Replacement Therapy;

ARDS, Acute Respiratory Distress Syndrome;

GCS Score, Glasgow Coma Scale Score;

Supplementary Table S2. Univariate and Multivariate Analysis Results

| Variable | Univariate analysis | | Multivariate analysis | |
| --- | --- | --- | --- | --- |
|  | OR 95%CI | P value | OR 95%CI | P value |
| Sofa Score | 1.25 (1.03-1.52) | 0.026 | 1.18 (0.92-1.51) | 0.198 |
| PO2/FiO2 | 0.98 (0.97-0.99) | 0.009 | 0.99 (0.98-1.01) | 0.423 |
| Diabetes | 4.12 (0.97-17.52) | 0.055 | 3.24 (0.58-18.12) | 0.181 |
| Tumor | 0.06 (0.01-0.48) | 0.009 | 0.15 (0.02-1.32) | 0.089 |
| TNF-α | 2.05 (1.01-4.16) | 0.047 | 1.62 (0.71-3.70) | 0.251 |

Supplementary Table S3. Detailed LC-MS Identification Parameters for the Five Key Serum Metabolite Biomarkers.

| Metabolite  Name | Ion Mode | Measured  m/z | RT^1^  (min) | MS/MS  Score2 | HMDB | KEGG | Mass Error  (ppm)^3^ | Confidence  Level^4^ |
| --- | --- | --- | --- | --- | --- | --- | --- | --- |
| Sebacic acid | Negative | 201.11 | 3.24 | 0.93 | HMDB0000792 | C08277 | <10 | Level 2 |
| Acylcarnitine 10:2 | Positive | 312.22 | 3.12 | 0.88 | Not Available | C02301 | <10 | Level 2 |
| Methyl acetate | Negative | 119.03 | 1.31 | 0.85 | HMDB0031523 | C17530 | <10 | Level 2 |
| Threonic acid | Negative | 135.03 | 0.85 | 0.84 | HMDB0000943 | C01620 | <10 | Level 2 |
| 1-RDN | Positive | 257.11 | 2.29 | 0.83 | HMDB0011648 | C15497 | <10 | Level 2 |

Footnotes:

^1^ RT: Retention Time

¹ MS/MS Score: Cosine similarity score (0-1) between the experimental and reference MS/MS spectrum from the in-house library. A score > 0.70 indicates a high-confidence match.

² Mass Error: The absolute difference between the measured m/z and the theoretical m/z from the database, expressed in parts per million (ppm). A threshold of < 10 ppm was applied for initial annotation.

³ Confidence Level: Level 2 (Putative Annotation), as defined by metabolomics standards. Identification is based on matching both accurate mass and MS/MS spectrum, but not confirmed with an authentic standard.

Supplementary Table S4. Clinical information of study cohorts Results of molecular docking of differential metabolites to disease-related proteins.

| Metabolite | Disease-related protein | Binding Affinity(kcal/mol) |
| --- | --- | --- |
| 1-RDN | ACADL | -6.9 |
| 1-RDN | AMPK | -5.3 |
| 1-RDN | L-FABP | -7.1 |
| 1-RDN | MCAD | -7.4 |
| 1-RDN | PAH | -7.9 |
| 1-RDN | PPARα | -6.1 |
| 1-RDN | TGF-β | -5.9 |
| Acylcarnitine 10:2 | ACADL | -4.6 |
| Acylcarnitine 10:2 | AMPK | -3.9 |
| Acylcarnitine 10:2 | L-FABP | -5.2 |
| Acylcarnitine 10:2 | PAH | -5.7 |
| Acylcarnitine 10:2 | PPARα | -5.6 |
| Acylcarnitine 10:2 | TGF-β | -4.4 |
| Acylcarnitine 10:2 | MCAD | -4.9 |
| Methyl_acetate | ACADL | -3.4 |
| Methyl_acetate | AMPK | -2.7 |
| Methyl_acetate | L-FABP | -3.1 |
| Methyl_acetate | PAH | -3.5 |
| Methyl_acetate | PPARα | -3.3 |
| Methyl_acetate | TGF-β | -3.2 |
| Methyl_acetate | MCAD | -3.8 |
| Sebacic_acid | ACADL | -5.8 |
| Sebacic_acid | AMPK | -3.9 |
| Sebacic_acid | L-FABP | -6.2 |
| Sebacic_acid | MCAD | -5.2 |
| Sebacic_acid | PAH | -6.2 |
| Sebacic_acid | PPARα | -6.0 |
| Sebacic_acid | TGF-β | -5.6 |
| Threonic_acid | ACADL | -5.4 |
| Threonic_acid | AMPK | -4.1 |
| Threonic_acid | L-FABP | -4.3 |
| Threonic_acid | MCAD | -4.7 |
| Threonic_acid | PAH | -5.0 |
| Threonic_acid | PPARα | -4.9 |
| Threonic_acid | TGF-β | -4.0 |

1. RDN, 1-(β-D-Ribofuranosyl)-1,4-dihydronicotinamide; ACADL, Acyl-CoA Dehydrogenase Long Chain; AMPK, AMP-Activated Protein Kinase; L-FABP, Liver-type Fatty Acid Binding Protein; MCAD, Medium-Chain Acyl-CoA Dehydrogenase; PAH, Phenylalanine Hydroxylase; PPARα, Peroxisome Proliferator-Activated Receptor Alpha; TGF-β, Transforming Growth Factor Beta.

Supplementary Table S5. Multivariable Logistic Regression Analysis of the Metabolite-Based SVM Model and Clinical Parameters for SA-AKI Prediction

| Variable | Multivariate analysis | |
| --- | --- | --- |
|  | OR 95%CI | P value |
| SVM Model | 25.45 (15.72-48.92) | <0.001 |
| Sofa Score | 1.32(0.95-1.84) | 0.096 |
| PO2/FiO2 | 0.98 (0.96-1.00) | 0.080 |
| Diabetes | 1.45 (0.78-2.69) | 0.239 |
| Tumor | 0.29 (0.04-2.17) | 0.227 |
| TNF-α | 1.85 (0.36-9.44) | 0.461 |


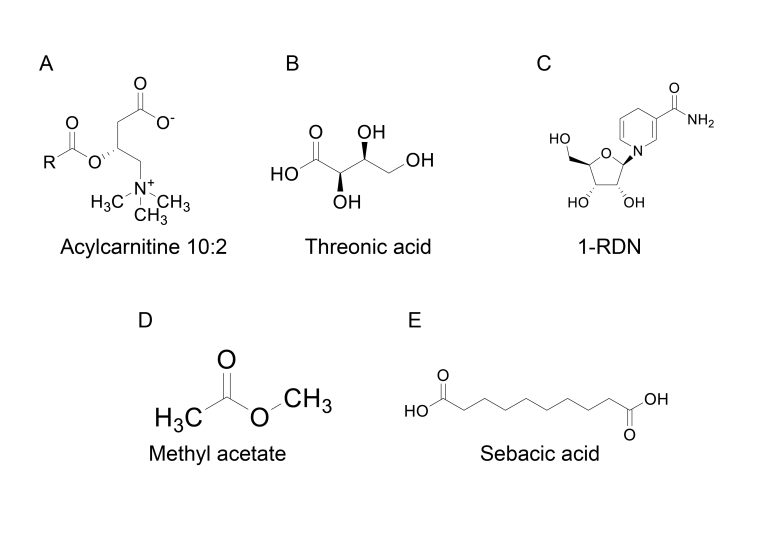


Supplementary Figure 1. A. Acylcarnitine 10:2; B. Threonic acid;C. 1-RDN; D.Methyl acetate; E. Sebacic acid


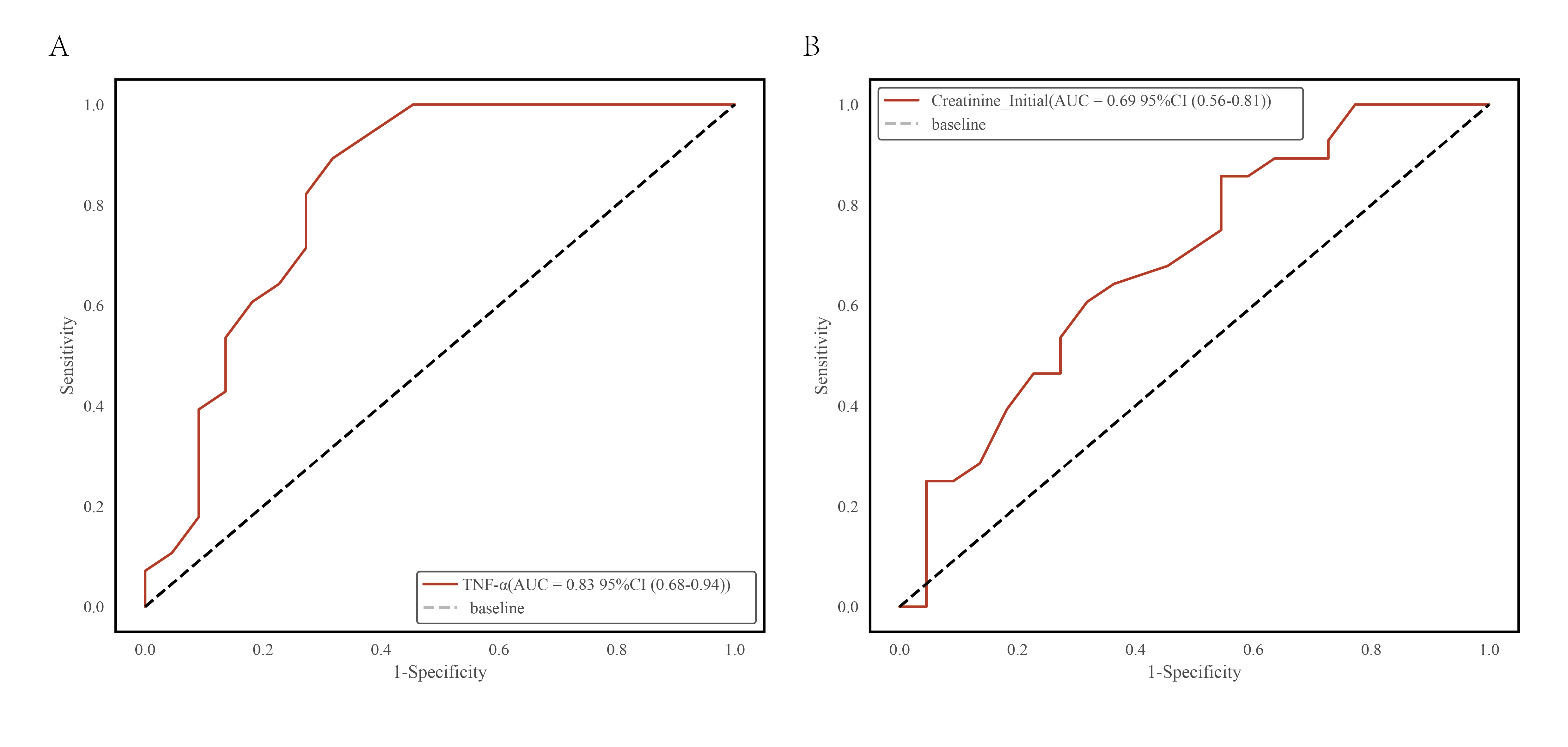


Supplementary Figure 2. A.The AUC values and ROC curves for TNF-alpha; B. The AUC values and ROC curves Creatinine_Initial.
